# Detection of incomplete atypical femoral fracture on anteroposterior radiographs via explainable artificial intelligence

**DOI:** 10.1038/s41598-023-37560-9

**Published:** 2023-06-27

**Authors:** Taekyeong Kim, Nam Hoon Moon, Tae Sik Goh, Im Doo Jung

**Affiliations:** 1 Department of Mechanical Engineering, Ulsan National Institute of Science and Technology, Ulsan, 44919 Republic of Korea; 2 Department of Orthopaedic Surgery, Biomedical Research Institute, Pusan National University Hospital, Pusan National University School of Medicine, Busan, 49241 Republic of Korea

**Keywords:** Computational biology and bioinformatics, Machine learning

## Abstract

One of the key aspects of the diagnosis and treatment of atypical femoral fractures is the early detection of incomplete fractures and the prevention of their progression to complete fractures. However, an incomplete atypical femoral fracture can be misdiagnosed as a normal lesion by both primary care physicians and orthopedic surgeons; expert consultation is needed for accurate diagnosis. To overcome this limitation, we developed a transfer learning-based ensemble model to detect and localize fractures. A total of 1050 radiographs, including 100 incomplete fractures, were preprocessed by applying a Sobel filter. Six models (EfficientNet B5, B6, B7, DenseNet 121, MobileNet V1, and V2) were selected for transfer learning. We then composed two ensemble models; the first was based on the three models having the highest accuracy, and the second was based on the five models having the highest accuracy. The area under the curve (AUC) of the case that used the three most accurate models was the highest at 0.998. This study demonstrates that an ensemble of transfer-learning-based models can accurately classify and detect fractures, even in an imbalanced dataset. This artificial intelligence (AI)-assisted diagnostic application could support decision-making and reduce the workload of clinicians with its high speed and accuracy.

## Introduction

Atypical femoral fractures occur in patients who have excessive femoral bowing or a history of long-term use of bisphosphonates for osteoporosis treatment. These types of fractures take the form of incomplete fractures, such as stress fractures, for a certain period before developing into complete fractures. In the early stage of an atypical femoral fracture, a micro-fracture is followed by healing. This process repeats and eventually produces cortical buckling in the lateral cortex of the femur. However, non-orthogeriatric clinicians are often unaware of the clinical implications of cortical buckling, and orthopedic surgeons can miss this lesion. This kind of lesion can be detected through the characteristic uptake in bone scans and bone marrow edema in magnetic resonance images (MRIs)^1^, but there is still a high probability of the lesion being misdiagnosed as a spine or knee joint pathology. The misdiagnosis or missed diagnosis can cause unnecessary or delayed treatment that results in a complete fracture.

As atypical femoral fractures are caused by the suppression of bone turnover, it has been theorized that delayed union and nonunion may occur because of a reduction in osteoblast and osteoclast activity^2^. If an incomplete fracture progresses to a complete fracture, more effort is required to heal the fracture. Therefore, detecting cortical buckling and performing preventive treatment are vital for improving clinical results.

To reduce diagnostic error and the subsequent personal and financial hardship, artificial intelligence (AI) has recently been used to provide a second opinion. As AI can optimize and monitor the process cost-effectively, it can be used in various fields such as manufacturing^3,4^, material engineering^5^, and thermal engineering^6^, along with digitalization^7^. In addition, the convolutional neural network (CNN), which is widely used in object detection and classification, has been used in diagnosis owing to its ability to detect complex patterns and extract relevant information from images. For example, Sannasi et al. proposed a deep CNN-based framework for the early diagnosis of breast cancer, which achieved a classification accuracy of 97.93%^8^. Wang et al. detected and classified mandibular fractures with an accuracy above 90% by applying a deep CNN to computed tomography (CT) scans^9^. Chung et al. also demonstrated the ability of AI to detect proximal humerus fractures with a top accuracy of 96%^10^. Furthermore, various studies have been conducted utilizing CNN models to detect fractures through radiography^11–14^. Raisuddin et al. developed a wrist fracture detection system based on CNN, achieving an impressive AUC of 0.99^11^. In addition, the system allowed for the localization of wrist fractures using Grad-CAM. Similarly, Murphy et al. developed a CNN-based system for detecting hip fractures, utilizing GoogleNet and achieving an accuracy rate of 92%^12^. The application of CNN models has also been extended to detecting rib and foot fractures accurately, leading to improved detection accuracy and efficiency for clinicians^13,14^.

However, if the dataset is too small to obtain enough features, there is a possibility of overfitting, which would result in poor performance. To overcome this limitation, a pre-trained network is generally employed for transfer learning, and this approach achieves excellent performance on small datasets. Hall et al. classified Covid-19 with an accuracy of 89.2% from a chest radiograph dataset that included 135 Covid-19 cases and 320 non-Covid-19 pneumonia cases^15^. Singh et al. used transfer learning to detect rare genetic diseases, such as Down’s syndrome. Their dataset originally comprised 1089 images and 12 classes, and after augmentation and transfer learning, the model showed a top accuracy of 97.66%^16^. Furthermore, AI has been applied to small datasets to detect abnormalities, such as tumors^17,18^, has demonstrated efficiency in supporting diagnosis from radiographs. The ensemble method was also applied to improve the accuracy^19^, which has better performance than a single classifier^20^.

Research on utilizing AI for diagnosing AFF has been conducted extensively. Zdolsek et al. employed transfer learning techniques, incorporating models such as ResNet50 and VGG19, achieving an impressive AUC accuracy of 0.94 for classifying normal femur factors (NFF) and AFF^21^. Similarly, other studies have successfully improved diagnostic accuracy, attaining an accuracy rate of 94.4% using transfer learning with models like VGG19^21^. While these studies have demonstrated high accuracy in classifying complete AFF from NFF, it is crucial to diagnose AFF accurately, even in its early and incomplete stages. To address this task, in this work, we adopted the transfer learning approach employed in previous studies, along with ensemble methods. Additionally, while techniques such as under-sampling and oversampling have been commonly applied to deal with imbalanced class data^12,22^, this work aimed to evaluate the performance of AI when sufficient features for each class were provided through preprocessing, eliminating the need for sampling techniques.

This study investigates the feasibility of using a machine-learning algorithm for the detection of incomplete atypical femoral fracture on an anteroposterior (AP) radiograph of the femur. First, we apply the Sobel filter to the dataset to clarify the edge of the bone, and then we train the transfer-learning-based CNN model using an ensemble method combining multiple models. Fracture localization is implemented using score-weighted class activation mapping (Score-CAM) technology, which represents regions that are relevant to a given class.

## Materials and methods

### Selection of fracture group and normal group

The study was conducted in accordance with the tenets of the Declaration of Helsinki and the protocol was approved by the institutional review board of Pusan National University Hospital with the number of 2209-032-119. To collect data, a review of the medical records of the two tertiary hospitals was conducted between January 2010 and December 2019. Informed consent was obtained from all patients. Incomplete atypical femoral fracture was defined in the following cases: (1) a distinct lateral cortical buckling without a history of trauma was confirmed by a radiologist and orthopedic surgeon; (2) the uptake of lateral cortical buckling was confirmed on the bone scan; and (3) bone marrow edema was confirmed in the MRI evaluation.

All femur radiographs were interpreted by a board-certified musculoskeletal radiologist who had 15 years of experience and an orthopedic surgeon who had 15 years of experience. The fracture group comprised 100 patients with incomplete atypical femoral fracture (mean age: 67.5 years; age range: 57–87 years); there were 96 women and 1 man. The radiographs for the femur group included 61 right femurs and 39 left femurs. In the normal group, all AP radiographs of the right and left femur were collected if there were no morphological abnormalities. Patients having neoplasms, significant deformities, or radiologic evidence of prior fracture or surgery were excluded. The normal group comprised 950 patients (498 women and 452 men) who had normal femur radiographs (mean age: 45.2 years; age range: 21–95 years). These included 400 right femurs and 550 left femurs.

A total of 1050 radiographs were obtained from the subject groups; this total included 950 normal and 100 atypical femoral fractures. The dataset was randomly split into a training set and a validation set, where 60% of the data were included in the training set (i.e., 570 normal and 60 atypical femoral fracture images).

Depending on the degree, incomplete atypical femoral fractures may not be noticeable, and the failure to promptly diagnose these fractures can result in complete fractures in the future. The objective of this study is to accurately detect and treat atypical femoral fractures in their early stages. To achieve this, a specific type of fracture that includes focal cortical changes was added to the dataset, targeting cases that clinicians are likely to misdiagnose. However, to demonstrate the robustness of our model, a test set was created by collecting atypical femoral fracture radiographs from various search engines, including Google. The test set comprises 20 normal cases and 20 fracture cases, with variations in resolution, size, and degree of fracture.

### Data preprocessing and augmentation

**Figure 1:**
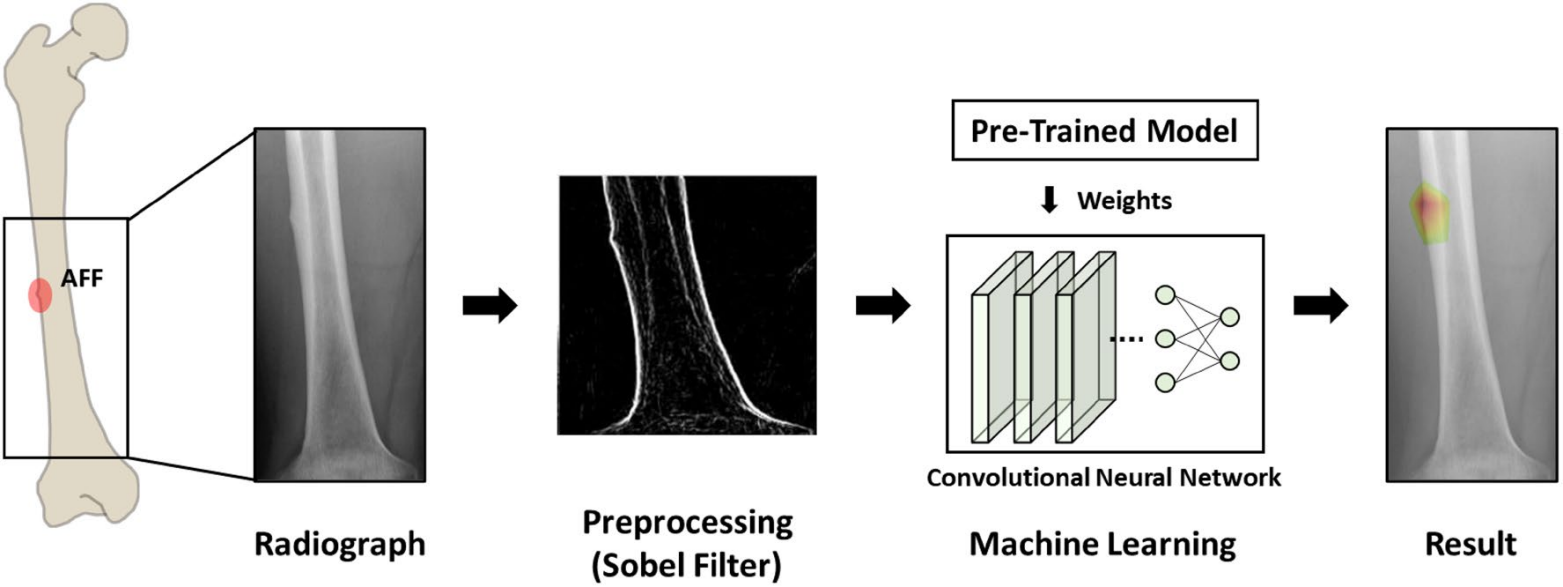
Schematic diagram of fracture detection from the radiograph.

As shown in Fig. 1, the region above the lesser trochanter and below the nutrient foramen was removed from the image, which is centered on the diaphysis (shaft) where the atypical femoral fracture occurs. In addition, the images were adjusted to 224 × 224 pixels to correspond to the input size of the model, and a Sobel filter^23^ was used to obtain the contours of the bones.

To prevent overfitting to the training set and ensure data diversity, we applied an image data generator to the preprocessed training images, which improved the model performance. The hyperparameters for zoom range and shear angle were set so that images were randomly zoomed between 80% (zoom in) and 120% (zoom out), and randomly sheared at an angle between − 0.2° and 0.2°. Additionally, horizontal and vertical flips were randomly employed to minimize the distinction between the two sides of the femur. Finally, the pixel values of the training and testing sets were rescaled from 0–255 to 0–1 bits.

### Model structure and evaluation

**Figure 2:**
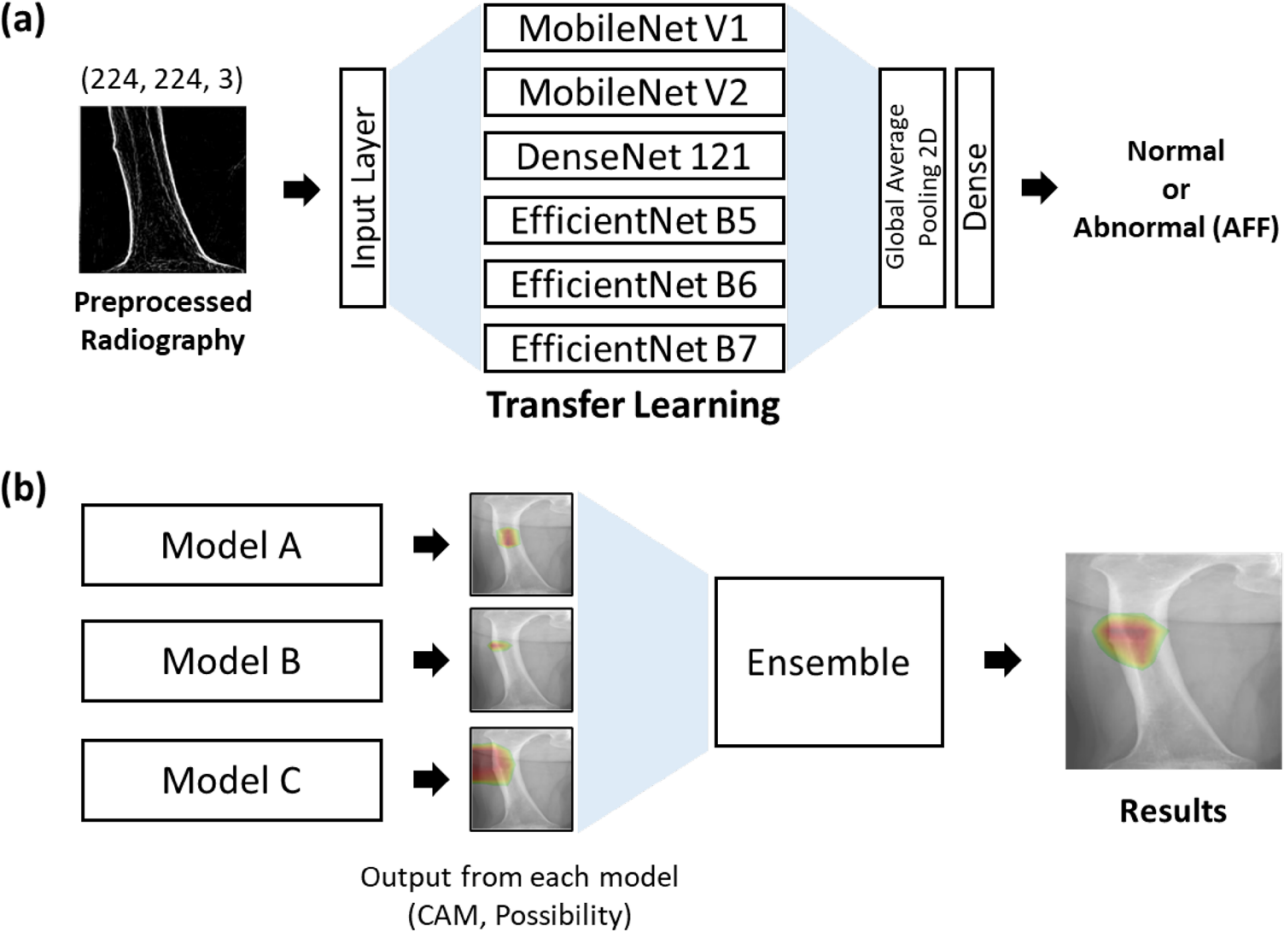
Schematic diagram of (**a**) transfer learning and (**b**) ensemble method. Transfer learning is performed for each of the six pre-trained models, and the three models (models a, b, and c) having the highest accuracy are selected for the ensemble.

A CNN was used to perform the classification and localization of atypical femoral fractures. In the case of fracture detection, there are two approaches: classification and identification of the region the model is looking at while classifying, and object detection, which aims to localize the fracture locations^24^. In this work, we employed the former method, the classification-based approach, which has also been widely utilized in previous fracture detection studies^25^. As shown in Fig. 2a, to obtain an accurate and efficient training model, we applied transfer learning, which imports model structures and weights from models pretrained on the ImageNet database^26^. The imported models were MobileNet V1^27^, MobileNet V2^28^, DenseNet 121^29^, and EfficientNet B5, B6, and B7^30^, which are widely used for their high accuracy in classification problems^31–33^. MobileNet successfully reduces model size and simplifies its structure using depth-wise separable convolution. DenseNet 121 also has a small number of parameters, and shows good predictive performance in solving the vanishing gradient problem. In addition, EfficientNet shows superior predictive performance by systematically scaling the network depth, width, and resolution. We imported these models and retained all the pre-trained layer weights.

The input image size of the model was $224 \times 224 \times 3$, and the batch size was 8. The maximum number of training epochs for each model was initially set to 200 epochs, and an early stopping callback terminated the training process when there was no improvement in the validation loss after more than 35 epochs. To monitor the convergence during model training, sparse categorical cross-entropy was used as a loss function. For the output layer, softmax was employed as an activation function to define the output that corresponded to the given input. Adam^34^, an optimization algorithm, was applied with an initial learning rate of 10^−4^, and the learning rate was decreased by 10^−6^ every 10 epochs.

The machine learning algorithms used in this work are based on Python 3.7 and TensorFlow 2, along with other libraries. In addition, ImageDataGenerator from Keras was used for radiograph preprocessing, and Keras applications were utilized for importing pre-trained models. The models were trained on a Ubuntu 18.04 server equipped with an AMD Ryzen Threadripper 3990X central processing unit (CPU) and an NVIDIA RTX 3090 graphics processing unit (GPU). After all models were trained, the three models having the highest accuracy, and the top five models in terms of accuracy were selected to utilize the ensemble method, which is a more reliable and more robust method than a single classifier (Fig. 2b). An odd number of trained models was chosen to prevent tie cases during voting.

The performance of the trained models was evaluated using the receiver operator characteristic (ROC) curve and area under the curve (AUC)^35^. The ROC curve consists of the true positive rate (sensitivity) and false positive rate (1—specificity), and the AUC shows the summary of the ROC curve. Here, an AUC of 1 indicates that the trained model predicts fracture perfectly on the radiograph, whereas an AUC of 0.5 indicates a random guess, and an AUC of 0 indicates totally incorrect predictions. In addition, the F1 Score, which is calculated by recall and precision, was also used to clearly evaluate the performance of the model in data class imbalances.

### Fracture localization

In diagnosing fractures using AI, the localization of fractures is an important task to support accurate interpretation. To visualize the evidence that the model recognized the fracture site, we used Score-CAM^36^, which is a CAM-based method for creating a heatmap on the radiograph by interpreting the CNN filter.

During the application of Score-CAM, activation maps are extracted during the first phase, where each activation acts as a mask on a raw image. Subsequently, its forward-passing score corresponding to the target class is obtained. In the second phase, the first phase is repeated for the number of activation maps. The results are obtained using a linear combination of score-based weights and activation maps.

The areas identified as containing fractures were marked by superimposing the CAM of the last convolution layer of the imported models on the original radiograph. We customized the heatmap to reveal only the upper 40% of the confidence level in a localized part. For the ensemble case, heatmaps from each model were superimposed, and the average of each pixel’s data was calculated. Finally, a heat map was applied to the calculated data.

### Ethical approval

All procedures performed in studies involving human participants were in accordance with the ethical standards of the institutional and/or national research committee and with the 1964 Helsinki declaration and its later amendments or comparable ethical standards. The current research was approved by the institutional review board of Pusan National University Hospital with the number of 2209-032-119.

## Results

**Figure 3:**
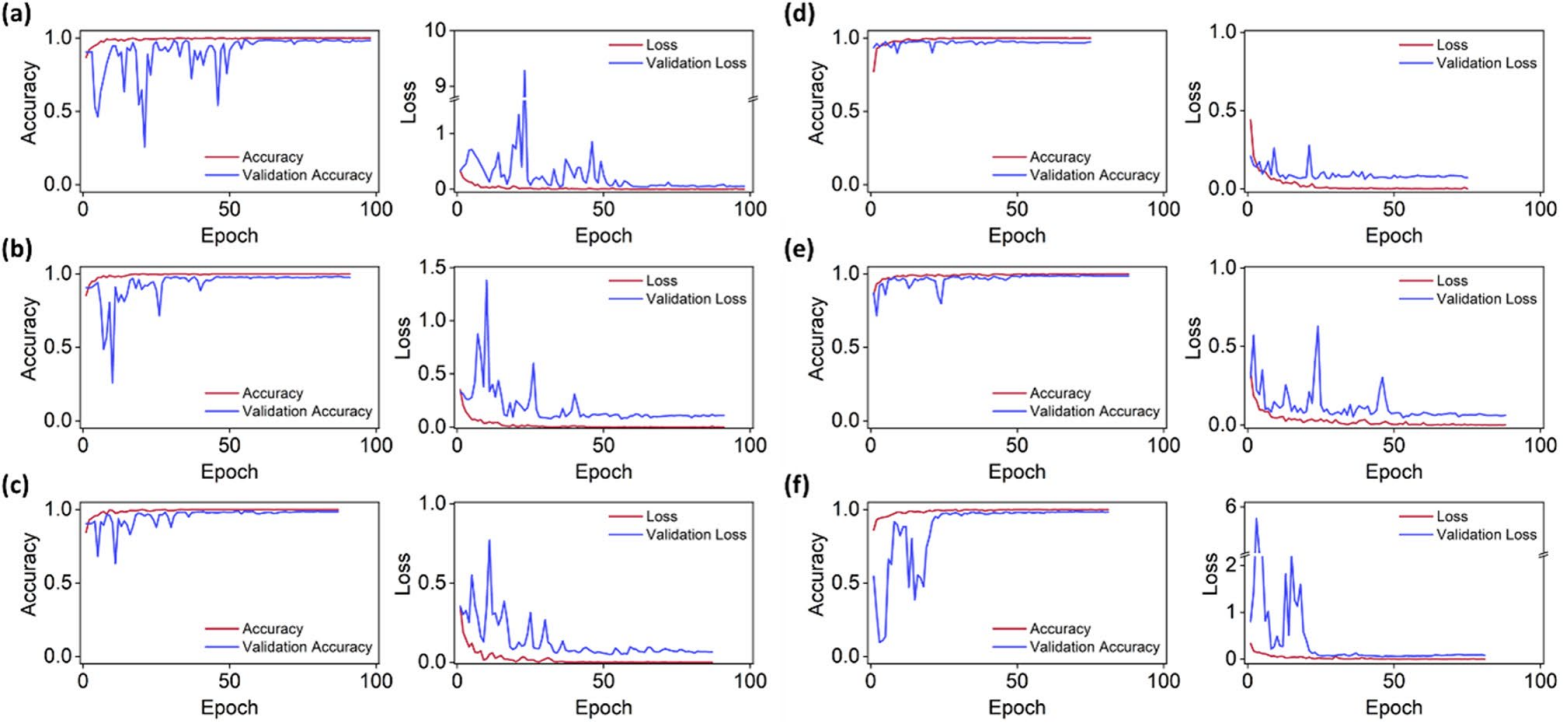
Accuracy and loss graphs for (**a**) EfficientNet B5, (**b**) EfficientNet B6, (**c**) EfficientNet B7, (**d**) DenseNet 121, (**e**) MobileNet V1, and (**f**) MobileNet V2. The red lines indicate the result for the training set, and the blue lines indicate the result for the validation set.

As shown in Fig. 3, the training of each model was stopped early and completed within 100 epochs, and overfitting was prevented. Each model took a few seconds per epoch for training, and a total of 20 to 30 min to complete the training. In proportion to the number of parameters, the learning time per epoch also increased. EfficientNet B5, B6, and B7, which have a large number of parameters, took 12, 14, and 18 s per epoch, respectively, whereas DenseNet 121 and the two MobileNets, which have a small number of parameters, took 6 and 5 s per epoch, respectively.

The learning accuracy of all models used for transfer learning converged to 1, and the loss converged to between 0 and 0.001. For the validation set, the models EfficientNet B7, DenseNet 121, and MobileNet V1 exhibited stable curves and rapidly converged to the optimal loss. However, in the case of DenseNet 121, which completed learning with the fewest epochs, the training and validation accuracies were relatively lower than those of the other models. Unlike the aforementioned models, EfficientNet B5, which began to converge after 50 epochs, showed the highest AUC value as a single classifier by achieving the optimal loss. MobileNet V2 achieved a minimum validation loss of 0.0509 after 46 epochs, which was 22% larger than the loss of EfficientNet B5, which showed the lowest validation loss. The optimal validation losses for each model were 0.0416, 0.0778, 0.0490, 0.0614, 0.0478, and 0.0509 (EfficientNet B5, EfficientNet B6, EfficientNet B7, DenseNet 121, MobileNet V1, and MobileNet V2, respectively), where EfficientNet B5 displayed the lowest value.

**Figure 4:**
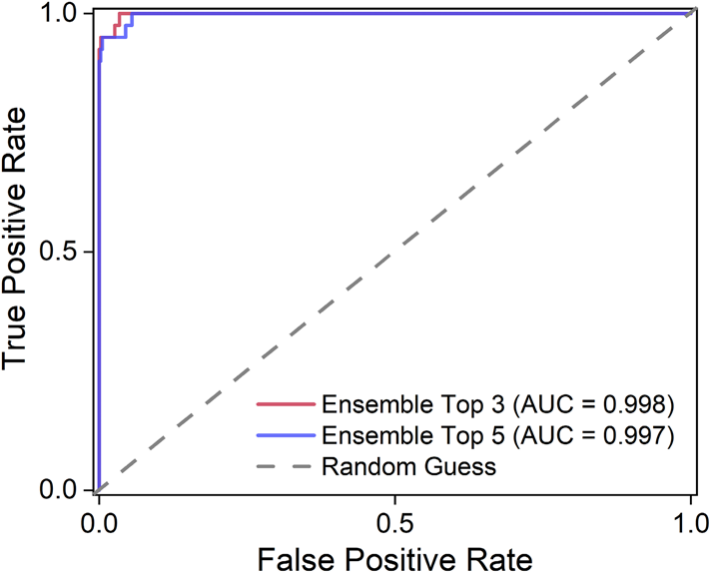
Training result of the models ROC curve and its AUC for the ensemble method.

The models were selected in terms of validation accuracy, as shown in Table 1, for the application of the ensemble method. MobileNet V1, EfficientNet B7, and B5, which achieved accuracies of 98.810, 98.810, and 98.571, respectively, were selected for the Ensemble Top 3 case. For the Ensemble Top 5, MobileNet V2 and EfficientNet B6, which achieved accuracies of 98.095 and 97.619, respectively, were added to the model for the Ensemble Top 3. The ROC curves of these two ensemble cases are shown in Fig. 4. The AUC of the Ensemble Top 3was 0.998, which was higher than the AUC of the Ensemble Top 5(0.997). In addition, for a single model, the F1 score was from 0.857 to 0.937 but the ensemble improved it up to 0.962.

**Table 1:** Accuracy and confusion matrix (TP, TN, FP, and FN) for each model.

Model	Accuracy (%)	Precision	Recall	F1 score	AUC	TP	TN	FP	FN
MobileNet V1	98.810	0.949	0.925	0.937	0.991	37	378	2	3
MobileNet V2	98.095	0.944	0.85	0.895	0.988	34	378	2	6
DenseNet 121	97.143	0.818	0.9	0.857	0.984	36	372	8	4
EfficientNet B5	98.571	0.905	0.95	0.927	0.992	38	376	4	2
EfficientNet B6	97.619	0.917	0.825	0.868	0.985	33	377	3	7
EfficientNet B7	98.810	0.949	0.925	0.937	0.993	37	378	2	3
Ensemble Top3	99.286	0.974	0.95	0.962	0.998	38	379	1	2
Ensemble Top5	99.048	0.974	0.925	0.949	0.997	37	379	1	3

The Ensemble Top 3 predicted normal radiographs with 99.9% accuracy and predicted atypical femoral fractures with 95% accuracy. However, Ensemble Top 5 showed 99.048% and 0.997 AUC, which are slightly lower accuracies than those of Top 3. Here, it can be seen that the Ensemble Top 3 model accurately predicted one more fracture radiograph than the Top 5 model. The accuracy and AUC values of each model for the validation set are shown in Table 1, where MobileNet V1 and EfficientNet B7 were the most accurate, with 98.810% accuracy and 0.960 AUC. It took 78.6 s to predict the fracture of the 420 radiographs included in the validation set using the Ensemble Top 3 model. The confusion matrix is also shown in Table 1 with true positive (TP), true negative (TN), false positive (FP), and false negative (FN) values.

**Figure 5:**
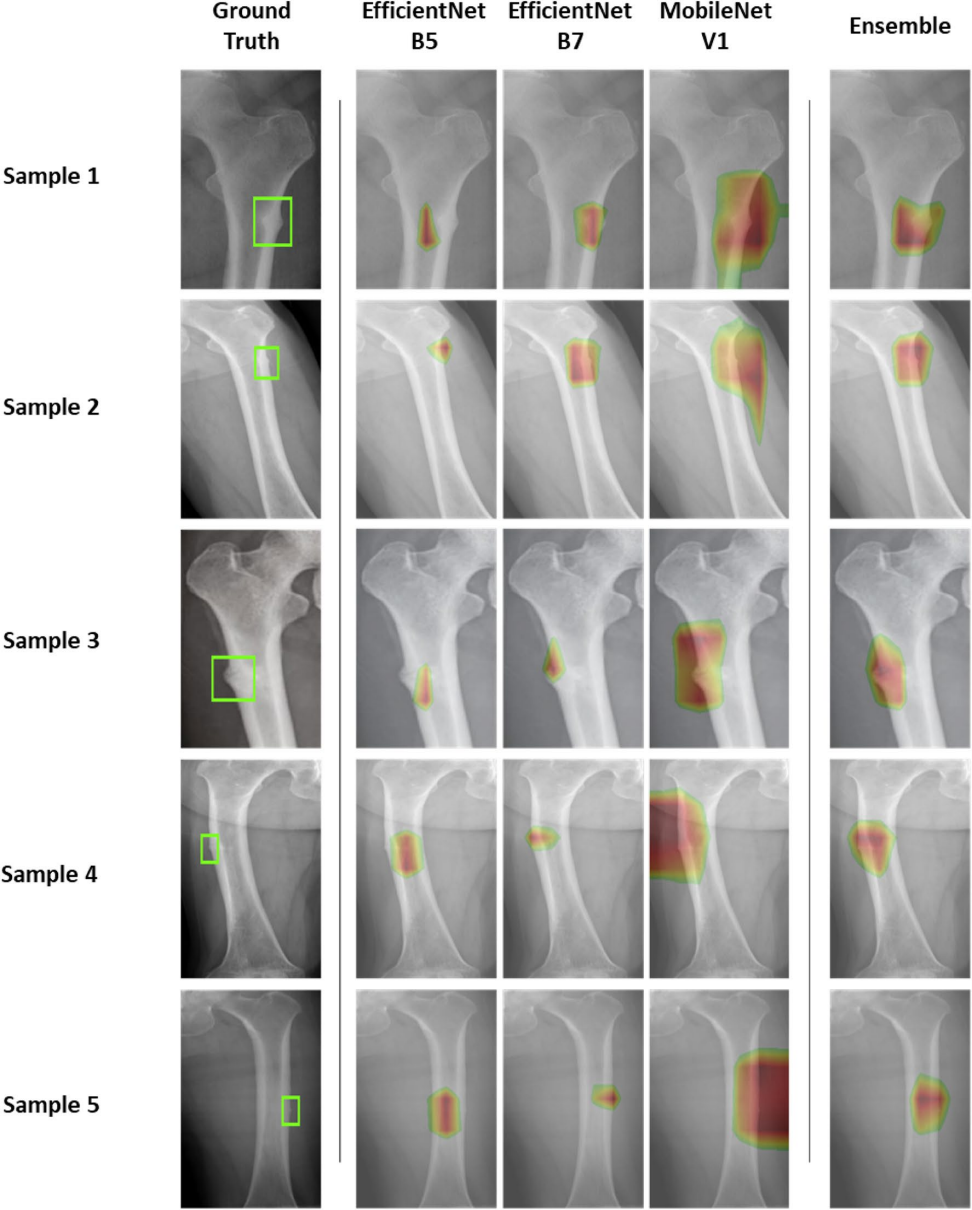
Five atypical femoral fracture predictions from the validation set. The green box indicates ground truth labeled by the radiologist. The results of three single classifiers (EfficientNet B5, B7, and MobileNet V1) and the ensemble results are represented.

Figure 5 shows images of the validation set with ground truth, which is an original radiograph labeled by a radiologist, and the prediction results. Ground truth, indicated by a green box, was not used for the training model; it was created for comparison with the predictive results. The localization of the fracture was performed by overlapping an original radiograph and the corresponding heatmap from the classifier using Score-CAM. The regions with greatest influence on the prediction results is marked in red, whereas regions having a lower influence are marked in green. Here, the confidence of each of the models EfficientNet B5, B7, and MobileNet V1, which compose Ensemble Top 3, is marked with a heatmap on an original radiograph. It can be seen that EfficientNet B5 shows a heatmap lightly shifted from the object, and MobileNet V1 shows a wide heatmap surrounding the object. Although EfficientNet B7 shows a narrow and exact confidence map, it often does not tend to cover the whole object, but we overcame this limitation by using the ensemble method. Detection and localization were accurately performed even for an almost invisible fracture, which is an early stage of atypical femoral fractures, as shown in the fifth sample. The probabilities of fracture on the five samples were 0.9994, 0.9477, 0.9997, 0.9988, and 0.9998 using Ensemble Top 3.

## Discussion

Object detection and feature extraction using AI have recently been developed in various fields. Utilizing such AI to substitute repetitive tasks that may cause human error and result in fatigue allows workers to achieve accuracy and efficiency in the work. Using AI to identify and classify atypical femoral fracture cases from radiographs can help in the early diagnosis of fractures and the timeliness of proper treatment, which is an important step in preventing unnecessary treatment and complete fractures. This study showed that the machine learning model could detect atypical femoral fractures with high accuracy, compared the performance of six different transfer learning models and applied the ensemble method to improve performance. In addition, the limitation of insufficient and imbalanced datasets, which causes a decline in the accuracy of a model, was overcome through data augmentation and the transfer of pre-trained weights from ImageNet.

Recently, the latest models have become increasingly complex, with a large number of parameters. However, using such deep and complex models with a small dataset can lead to inefficiency and inaccuracy as the models tend to overfit^37,38^. As shown in Table 2, we got similar results from employing relatively recent and complex models which were not selected and mentioned in method section. These findings support the notion that the latest and larger models do not consistently yield superior performance. Although the model we used is not the most up-to-date, previous studies have demonstrated its strong performance^39,40^, and it has shown similar performance on our dataset. Therefore, we anticipate that these results will serve as a guideline for selecting an appropriate model in cases where obtaining sufficient data, such as for rare diseases, is challenging.

**Table 2:** Comparison of various model performance.

Model	F1 score	Training time (s)	Number of parameters
EfficientNetB7 (This work)	0.937	13	66.7 M
ResNet152V2	0.838	7	60.4 M
EfficientNetV2M	0.894	7	54.4 M
EfficientNetV2L	0.911	13	119.0 M
ConvNeXtBase	0.174	12	88.5 M
ConvNeXtLarge	0.174	22	197.7 M

**Figure 6:**
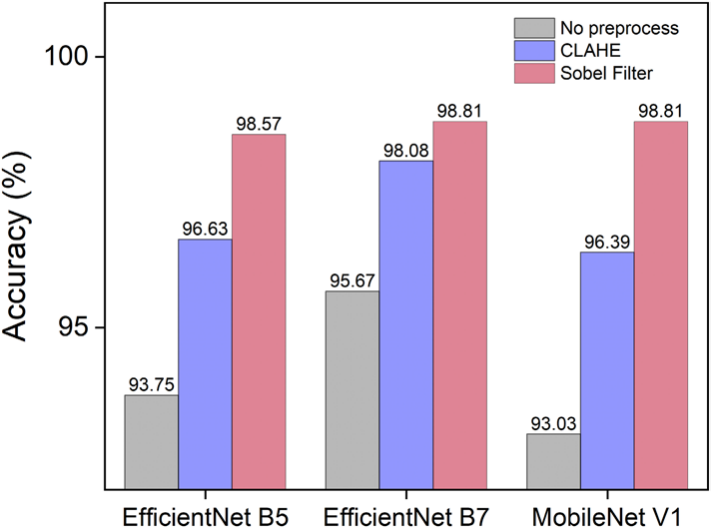
Comparison of accuracy based on the application of the Sobel filter and CLAHE. When the Sobel filter was applied to preprocess the radiographs, the accuracy was improved for EfficientNet B5, EfficientNet B7, and MobileNet V1 by 4.8%, 3.1%, and 5.8%, respectively, while CLAHE showed accuracy improvement of 2.9%, 2.4%, and 3.4%, respectively.

To observe the effect of the preprocessing, an ablation study was conducted not only for the Sobel filter but also for contrast-limited adaptive histogram equalization (CLAHE)^41^. Variables such as learning rate, epoch, and the data used for training was fixed, and the accuracy was compared when either the Sobel filter or CLAHE was applied or not. As shown in Fig. 6, by applying the Sobel filter to the radiographs, we were able to achieve up to a 5% improvement in accuracy for each model. This result indicates that the Sobel filter is more appropriate for our dataset compared to CLAHE.

We observed that an ensemble of a few accurate models led to more accurate results than an ensemble of many models including low prediction accuracy. In addition, when only 100 normal data points were used to resolve the data imbalance, the prediction was still accurate, but the CAM deviated significantly from the ground truth. However, using all the normal data (i.e., 950) resulted in an excellent match between the CAM and the ground truth, as shown in Fig. 5. The lightest of the models that we selected, MobileNet V1, continuously read 28 radiographs per second. In the case of the Ensemble Top 3 model, the three models read 5 radiographs per second, which reduced the speed relative to a single model, but the accuracy improved by approximately 1%. AFF, one of the rare diseases, led to a significant imbalance between data classes, making it difficult to determine a clear difference solely based on accuracy. However, the F1 Score showed that the ensemble model improved performance by up to 10.9% compared to a single model. Accordingly, it is obvious that speed of decision-making for diagnose by the model is much faster than that of a specialist, and this result shows that AI can assist specialists who are experiencing excessive workload and fatigue by suggesting reliable opinions and preventing misinterpretation.

**Figure 7:**
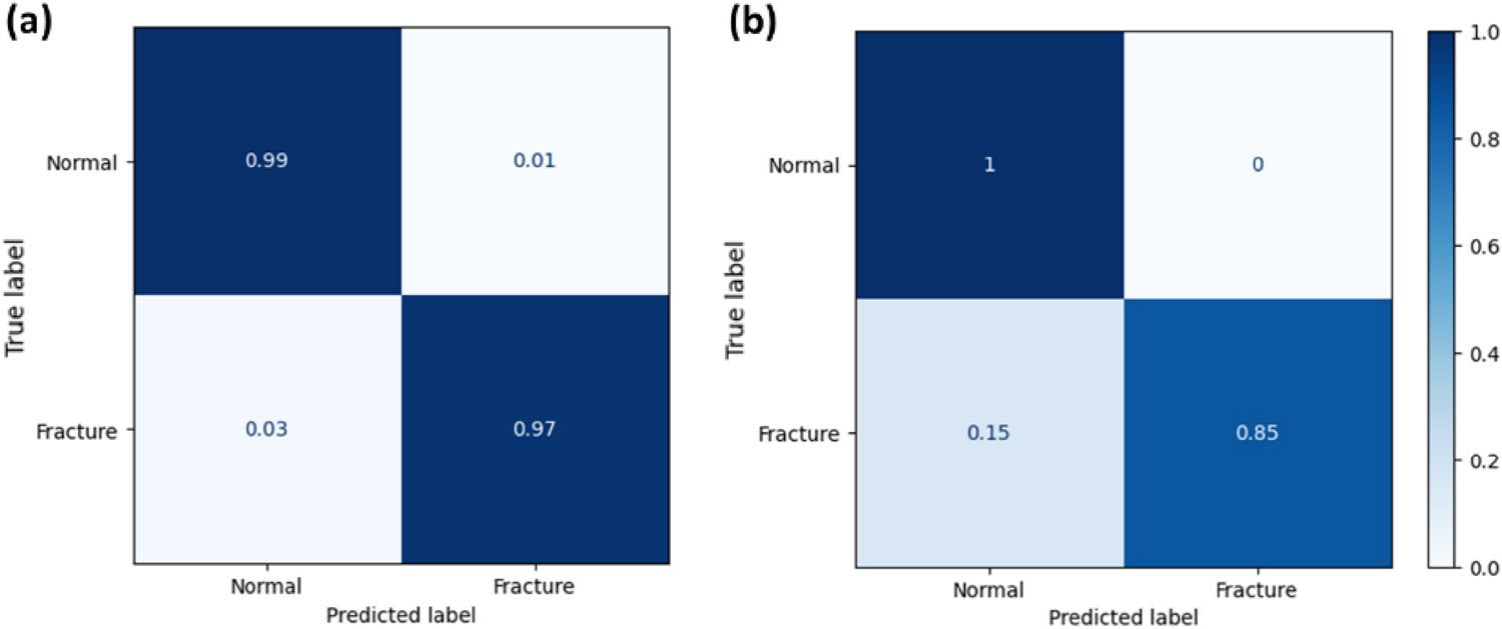
Confusion matrix for (**a**) validation set and (**b**) test set.

As shown in Fig. 7, our model achieved a 92.5% accuracy on the test set. Because some of the image sources in the test set contain distortions, the slightly lower defect prediction accuracy on the test set compared to the validation set. However, it is important to note that such problems are rarely encountered in hospital settings. Once the minimum resolution is ensured, our model demonstrates consistent prediction and localization performance across various sizes and degrees of fracture.

This study has some limitations. First, atypical femoral fracture is a rare type of fracture; even though all patients with incomplete atypical femoral fracture who had visited two hospitals in the past 10 years were reviewed, only 67 incomplete atypical femoral fractures were identified. Second, although lateral cortical buckling was accurately detected in this study, the fracture probability was not presented; thus, guidelines for treatment could not be offered. However, detecting a significant pathology that clinicians can easily overlook enables the establishment of an appropriate referral system in hospitals. In the case of spine or knee surgeons, our AI model provides an opportunity to focus on specialized care by R/O (rule out). Third, we did not evaluate exceptional situations, such as a femur with implants inserted or severe deformation. Therefore, further research is required to determine whether the detection of local buckling is possible where implants are inserted or severe deformation.

## Conclusion

Atypical femoral fractures were successfully detected via the developed approach of the transfer learning-based ensemble method. This study showed that fracture diagnosis with high accuracy using was possible with the use of transfer learning, even in rare cases for which balanced and sufficient data were not available. The prediction and localization results showed that the use of AI for fracture diagnosis has potential as a basis for specialist diagnosis. AI for fracture diagnosis could also be used effectively to train experts without incurring additional costs. Although our model was evaluated and used for atypical femoral fracture diagnosis, it is not limited to AP radiographs and can be applied to similar various localized radiographs.

## Data Availability

The datasets analyzed during the current study are not publicly available due to privacy restrictions, but are available to reviewers on reasonable request. Requests for data should be made to Prof. Nam Hoon Moon (ansskagns@daum.net) or Prof. Im Doo Jung (idjung@unist.ac.kr).
